# Ressincronização com Estimulação em Área de Ramo Esquerdo em Paciente Chagásico e Dependente da Estimulação Cardíaca Artificial: Relato de Caso

**DOI:** 10.36660/abc.20220464

**Published:** 2023-07-19

**Authors:** Raoni de Castro Galvão, João Paulo Velasco Pucci, Ofir Gomes Vieira, Edvagner Leite Sérgio de Carvalho, Winder Marconsini Soares, Guilherme Cunha dos S. Teles

**Affiliations:** 1 Centro de Ritmologia de Brasília Brasília DF Brasil Centro de Ritmologia de Brasília, Brasília, DF – Brasil; 2 Hospital de Base do Distrito Federal Brasília DF Brasil Hospital de Base do Distrito Federal, Brasília, DF – Brasil

**Keywords:** Cardiomiopatia chagásica, Estimulação Cardíaca Artificial, Terapia de Ressincronização Cardíaca

## Introdução

A estimulação monossítica persistente do ventrículo direito pode provocar efeitos deletérios na função ventricular.^1^ Pacientes com miocardiopatia chagásica (MCC) portadores de disfunção ventricular e que necessitam de estimulação artificial (ECA) podem acelerar a evolução para insuficiência cardíaca (IC) pela doença de base e a consequente dissincronia ventricular.

A terapia de ressincronização cardíaca (TRC) é uma alternativa consagrada na cardiologia há mais de 20 anos para tratamento da insuficiência cardíaca com fração de ejeção (FEVE) reduzida com distúrbio de condução intraventricular (BRE sobretudo).^2,3^ Entretanto, o efeito da TRC nos chagásicos pode não ser tão exuberante em relação a outras cardiopatias. O alto grau de fibrose miocárdica, sobretudo em parede lateral de ventrículo esquerdo (VE) ou outras regiões alvo para posicionamento do eletrodo, pode ser um dos fatores que justifiquem a resposta pouco animadora a TRC.^4^

Buscando uma estimulação mais fisiológica, na última década, intensificaram-se estudos de estimulação direta do sistema excito-condutor (His e ramo esquerdo (RE)) como alternativa à TRC convencional.^5^ Publicações recentes já mostram a similaridade e até superioridade dessa estimulação em relação à TRC tradicional.^6-13^ Sendo assim, essa modalidade vem se tornando uma alterativa real nesses pacientes. No entanto, não há conteúdo na literatura sobre a resposta da estimulação dita fisiológica em pacientes chagásicos.

Relatamos o caso de uma paciente com MCC e dissincronia ventricular, portadora de MP bicameral por bloqueio atrioventricular total (BAVT), submetida à TRC com estimulação em área de RE, apresentando boa resposta clínica e anatômica no seguimento.

## Relato de Caso

Mulher, 64 anos, com MCC crônica e IC. Apresentou BAVT em 2004. Implantado marca-passo bicameral. Trocado gerador em 2012 e 2019. Nesta última, implantado novo eletrodo atrial por fratura de anterior.

**Figura 1:**
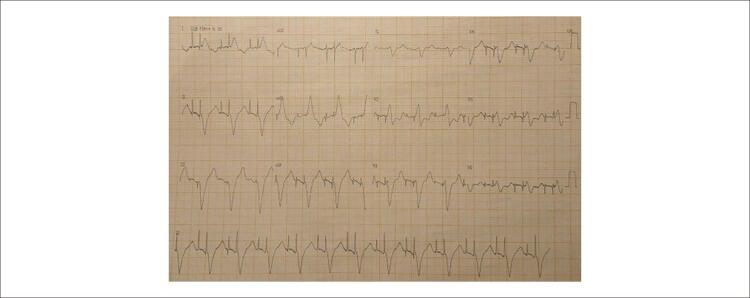
– Eletrocardiograma inicial, QRS 240 ms.

A paciente evoluiu com IC progressiva, mantendo classificação de NYHA III/IV. Entre 2019 e 2020 esteve por diversas vezes internada por IC descompensada, apesar de tratamento medicamentoso otimizado com metoprolol, losartana e espironolactona em doses máximas toleradas. Ecocardiograma transtorárico (ECOTT) de 08/2020, FEVE (Simpson) de 26%, volume atrial esquerdo, 37 ml/m^2^, diâmetro diastólico final do VE, 62 mm, movimento assincrônico septal, hipocinesia difusa, predomínio inferior e ínfero-lateral e aneurisma látero-basal; ECG com estimulo ventricular simulando BRE, QRS 240 ms e eixo QRS -60° (Figura 1).

Indicada TRC com estimulação septal IV profunda, pois havia o aneurisma látero-basal e a hipocinesia/acinesia ínfero-lateral que poderiam comprometer o sítio de posicionamento do eletrodo de VE em uma TRC tradicional. Os eletrodos antigos impossibilitaram uma melhor avaliação destas paredes por ressonância magnética cardíaca.

**Figura 2:**
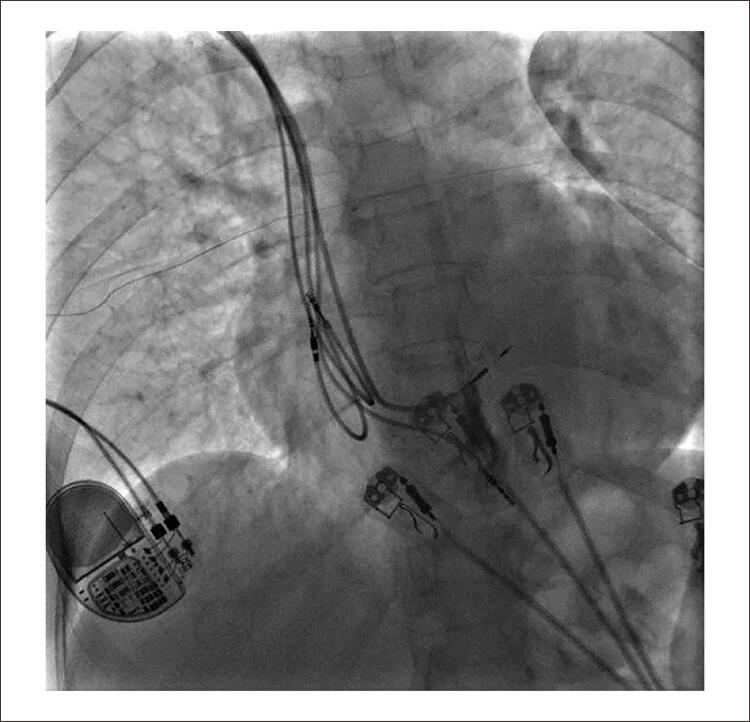
– Eletrodo em septo IV profundo (OAE 35°).

Em 09/2021, implantado eletrodo em septo IV profundo por punção venosa subclávia direita. Utilizados bainha C315his (Medtronic) e eletrodo C3830 (Medtronic) conectado a polígrafo multicanal. Após determinação do local do feixe de HIS, bainha avançada em 1,5 cm em direção ao ápice do VD, e inserido eletrodo novo em septo. Posição final confirmada após infusão de contraste iodado (Figuras 2 e 3). Ao término, foi programado TRC-p modo DDD 60bpm, intervalo AV 150 ms pace e 120 ms sense, intervalo VV com V(septo)-V(ponta) em 30 ms. ECG final: ritmo de marcapasso, estimulação atrioventricular, gerando QRS 125 ms, eixo -10° (figura 4). Limiar de comando de eletrodo septal profundo 0,8 V x 0,4 ms (uni) e impedância 475 ohm unipolar e 553 ohm bipolar. Não foram observadas dificuldades técnicas adicionais neste procedimento apesar de ser realizado pelo lado direito.

**Figura 3:**
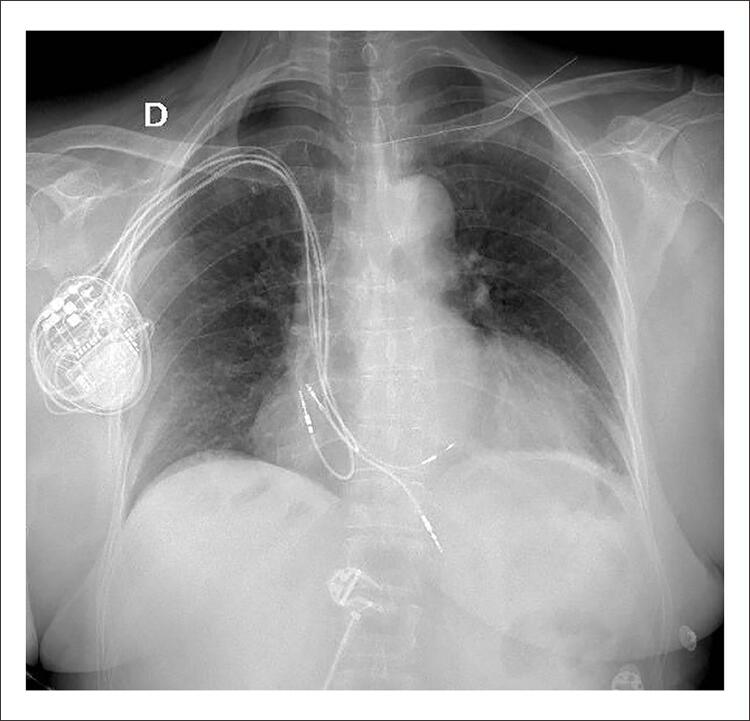
– Radiografia final pós-procedimento.

**Figura 4:**
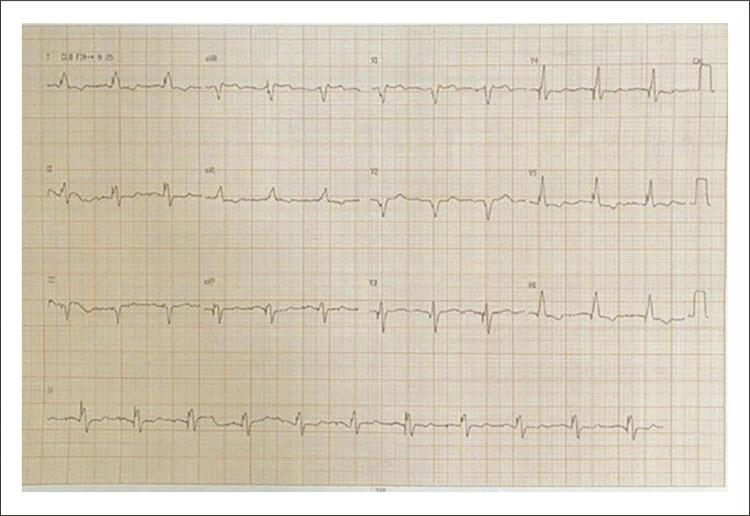
– Eletrocardiograma pós-estimulação septal profunda. Observa-se redução do QRS.

**Figura 5:**
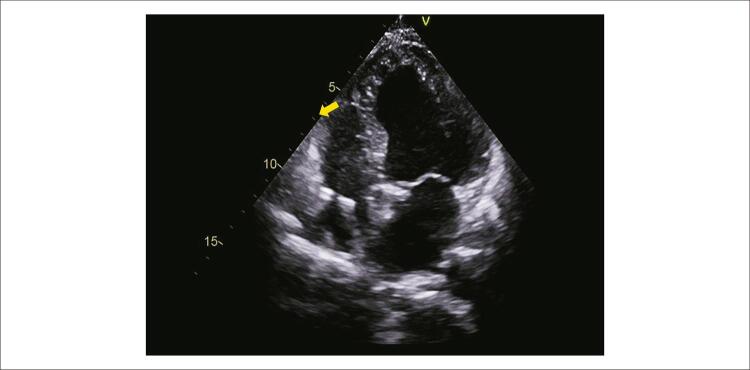
– Ecocardiograma mostrando ponta de eletrodo septal (seta).

No 1° mês pós-operatório, ECOTT com FEVE (38%), volume atrial esquerdo, 30 ml/m^2^, diâmetro diastólico do VE, 60 mm, sistólico, 41 mm, septo IV, 8 mm, e parede posterior, 7 mm, redução de dissincronia, mantendo alterações de contratilidade secundários a MCC (Figura 5). Além disso, apresentou melhora clínica importante para NYHA I.

Última avaliação em 05/2022, mantinha-se em NYHA I. Novo ECOTT com FEVE 38%, diminuição da dissincronia, mantidas alterações segmentares da contratilidade e aneurisma prévios e mantida a tendência de diminuição em câmaras esquerdas, volume atrial esquerdo, 25,1 ml/m^2^, diâmetro diastólico do VE, 57 mm, sistólico, 45 mm, septo IV, 9 mm, e parede posterior, 5 mm. Mantivemos tratamento otimizado para insuficiência cardíaca. Durante todo o seguimento pós-operatório, a paciente manteve as mesmas medicações de uso prévio nas mesmas doses.

## Discussão

Aproximadamente 20 a 30% dos pacientes submetidos à TRC tradicional são não respondedores. Em pacientes chagásicos, há poucos dados disponíveis na literatura, mostrando que a resposta nestes pode ser ainda pior. A fibrose miocárdica pode dificultar a resposta nestes pacientes, além de comprometer um local ótimo para posicionamento do eletrodo de VE em veia marginal de seio coronário.

Esta paciente apresentava alterações estruturais no VE, comprometendo o posicionamento de um eletrodo via seio coronário, nos motivando ao implante em septo IV profundo, visando estimulação de RE. A dissincronia gerada pela EA prévia seguramente colaborou para a deterioração clínica e nos motivou a realizar esta mudança para TRC, já que ela poderia ser corrigida com a estimulação de RE. Apesar do insucesso na estimulação seletiva de RE, conseguimos estimular uma área aliando ótimo limiar de comando e programação final do TRC-p, o que permitiu redução do QRS em quase 110 ms, normalizando o seu eixo, claramente promovendo uma estimulação mais rápida utilizando-se do sistema excito-condutor cardíaco original e reduzindo a dissincronia original.

Isto permitiu uma resposta clínica e anatômica rápida, já no 1º mês pós-operatório. Acreditamos que as alterações anatômicas de base (aneurisma látero-basal de VE e hipocinesia/acinesia em parede ínfero-lateral) impediram uma elevação ainda maior da FEVE.

A dissincronia gerada pela estimulação ventricular monossítica crônica, com QRS original alargado, pode ter sido responsável por parte da queda da FEVE nesta paciente. No entanto, obtivemos boa resposta da TRC mesmo em uma paciente chagásica com alterações anatômicas de base.

## Conclusão

Registros da resposta da TRC em chagásicos é escasso. Serão necessários estudos mais robustos para avaliar a resposta destes pacientes a TRC. Não há, até o momento, relatos de caso em MCC com respostas à TRC pela estimulação do sistema de condução, o que torna este caso pioneiro.

Com este relato, evidenciamos que a TRC com estimulação do RE com eletrodo em septo IV profundo é uma alternativa em pacientes chagásicos que cursam com queda da FEVE, sobretudo naqueles com dissincronia gerada pela ECA e que apresentam fibrose em região alvo de implante de eletrodo de VE na TRC tradicional.
