# Supplementary material for: Ressincronização com Estimulação em Área de Ramo Esquerdo em Paciente Chagásico e Dependente da Estimulação Cardíaca Artificial: Relato de Caso
Source: Arq Bras Cardiol. 2023 Jul 19;120(7):e20220464. [Article in Portuguese] doi: 10.36660/abc.20220464 (PMC10374266; doi:10.36660/abc.20220464)
Supplement: Translation

# Translation: Left Bundle Branch Area Pacing for Resynchronization in Chagasic Patient and Dependent on Artificial Cardiac Pacing. Case Report

**Authors:** Raoni de Castro Galvão, João Paulo Velasco Pucci, Ofir Gomes Vieira, Edvagner Leite Sérgio de Carvalho, Winder Marconsini Soares, Guilherme Cunha dos S. Teles

**Affiliations:** 1 Brazil Centro de Ritmologia de Brasília, Brasília, DF – Brazil; 2 Brazil Hospital de Base do Distrito Federal, Brasília, DF – Brazil

**Keywords:** Chagas Cardiomyopathy, Cardiac Pacing Artificial, Cardiac Resynchronization Therapy

## Introduction

Persistent monosytic pacing of the right ventricle can cause deleterious effects on ventricular function.^1^ Patients with Chagas cardiomyopathy (CCM) with ventricular dysfunction, requiring artificial heart stimulation (AHS) may accelerate the progression to heart failure (HF) due to the underlying disease and the consequent ventricular dyssynchrony.

For over 20 years, cardiac resynchronization therapy (CRT) has been an established alternative in cardiology for the treatment of heart failure with reduced ejection fraction (LVEF) with intraventricular conduction disorder (especially LBBB).^2,3^ However, the effect of CRT on Chagas patients may not be as clear in relation to other heart diseases. The high degree of myocardial fibrosis, especially in the left ventricle (LV) lateral wall or other target regions for electrode placement, may be one of the factors that would explain this poor response to CRT.^4^

The search for a more physiological stimulation has led studies of direct stimulation of the excito-conductor system (His and left branch (LB)) as an alternative to conventional CRT^5^to intensify over the last decade. Recent publications show the similarity and even superiority of this stimulation compared to traditional CRT.^6-13^ Therefore, this modality has become an actual alternative for these patients, despite the lack of literature on the so-called physiological stimulation response in Chagas patients.

We report the case of a patient with CCM and ventricular dyssynchrony, with dual-chamber pacemaker for complete atrioventricular block (CAVB), who underwent CRT with stimulation in the LB area, showing good clinical and anatomical response at follow-up.

## Case Report

Female, 64 years old, with chronic CCM and HF. The patient had CAVB in 2004, for which a dual-chamber pacemaker was implanted. The generator was replaced in 2012 and 2019, and in the latter, a new atrial electrode was implanted due to an anterior fracture.

The patient evolved with progressive HF, maintaining NYHA III/IV classification. Between 2019 and 2020, she was hospitalized several times for decompensated HF, despite optimized drug treatment with metoprolol, losartan and spironolactone at maximum tolerated doses. Transthoracic echocardiogram (TTE), August/2020, showed LVEF (Simpson) of 26%, left atrial volume, 37 ml/m^2^, LV end-diastolic diameter, 62 mm, asynchronous septal movement, diffuse hypokinesia, inferior and inferolateral predominance, and laterobasal aneurysm; ECG showed ventricular stimulus simulating LBBB, QRS 240 ms and -60° QRS axis (Figure 1).

**Figure 1:** – Initial electrocardiogram, QRS 240 ms.

CRT with deep IV septal stimulation was indicated, since the laterobasal aneurysm and inferolateral hypokinesia/akinesia could compromise the LV electrode placement site in a traditional CRT. The existing electrodes prevented a better evaluation of these walls by cardiac magnetic resonance imaging.

In 09/2021, an electrode was implanted in the deep IV septum via a right subclavian venipuncture. A C315his sheath (Medtronic) was used and a C3830 electrode (Medtronic) was connected to a multichannel polygraph. After determining the location of the HIS bundle, the sheath was advanced 1.5 cm towards the apex of the RV, and a new electrode was inserted in the septum. The end position was confirmed after infusion of iodinated contrast (Figures 2 and 3). Upon conclusion, a CRT-p DDD mode 60bpm was programmed, AV 150 ms pace interval and 120 ms sense, VV interval with V(septum)-V(tip) in 30 ms. Final ECG: Pacemaker pacing, atrioventricular stimulation, generating QRS 125 ms, -10° axis (Figure 4). Deep septal electrode command threshold 0.8 V x 0.4 ms (uni) and 475 ohm unipolar and 553 ohm bipolar impedance. No additional technical difficulties were observed during this procedure despite being performed on the right side.

**Figure 2:** – Electrode in deep IV septum (OAE 35°).

**Figure 3:** – Final post-procedure radiograph.

**Figure 4:** – Electrocardiogram after deep septal stimulation. QRS reduction is observed.

In the first postoperative month, TTE showed LVEF (38%), left atrial volume, 30 ml/m2, LV diastolic diameter, 60 mm, systolic, 41 mm, IV septum, 8 mm, and posterior wall, 7mm, dyssynchrony reduction, maintaining alterations in contractility secondary to CCM (figure 5). Additionally, the patient showed significant clinical improvement, being classified as NYHA I.

**Figure 5:** – Echocardiogram showing septal electrode tip (arrow).

Last evaluation in 05/2022, remained as NYHA I. New TTE with LVEF 38%, decreased dyssynchrony, maintenance of segmental changes in contractility and previous aneurysm, and the tendency of decrease in left chambers was maintained, left atrial volume, 25.1 ml/m2, LV diastolic diameter, 57 mm, systolic diameter, 45 mm, IV septum, 9 mm, and posterior wall, 5 mm. Optimized treatment for heart failure was maintained. During the entire postoperative follow-up, the patient maintained the same medications previously used at the same doses.

## Discussion

Approximately 20 to 30% of patients undergoing traditional CRT are non-responders. In Chagas patients, there is a lack of literature data available, showing that the response in these patients can be even worse. Myocardial fibrosis may hinder the response in these patients, in addition to compromising an optimal site for positioning the LV electrode in the marginal vein of the coronary sinus.

This patient showed structural alterations in the LV, compromising the electrode placement via the coronary sinus, which motivated us to implant it in the deep IV septum, aiming at LB stimulation. The dyssynchrony generated by the previous AS certainly contributed to the clinical deterioration and motivated us to perform this upgrade to CRT, since it could be corrected with LB stimulation. Despite the failure in the selective stimulation of the LB, we were able to stimulate an area combining an excellent command threshold and the final programming of the CRT-p, which resulted in a QRS reduction of almost 110 ms, a normalization of its axis, and a reduction of dyssynchrony caused by the original cardiac excito-conductive system.

This allowed for a quick clinical and anatomical response, as early as the first postoperative month. We believe that the baseline anatomical alterations (LV lateral-basal aneurysm and hypokinesia/akinesia in the inferolateral wall) prevented an even greater increase in LVEF.

It is possible that dyssynchrony caused by chronic monosytic ventricular pacing with widened QRS contributed to part of the drop in LVEF in this patient, but a good response to the CRT was obtained, despite being a Chagas patient with baseline anatomical abnormalities.

## Conclusion

Records of CRT response in Chagas patients are scarce. The response of these patients to CRT requires a range of more robust studies. So far, there are no case reports in CCM with responses to CRT by stimulation of the conduction system, which makes this case the first of its kind.

This report shows that CRT with LB stimulation by an electrode in the deep IV septum is an alternative for Chagas patients who experience an LVEF drop, especially those with dyssynchrony generated by AHS and those who have fibrosis in the target region of LV electrode placement implantation on traditional CRT.
